# A process evaluation of the improving wisely intervention: a peer-to-peer data intervention to reduce overuse in surgery

**DOI:** 10.1186/s12913-020-06017-4

**Published:** 2021-01-29

**Authors:** Christine Fahim, William E. Bruhn, John G. Albertini, Marty A. Makary

**Affiliations:** 1 Dept. of Health Policy & Management, Johns Hopkins University, Baltimore, MD USA; 2 Li Ka Shing Knowledge Institute, Unity Health Toronto, Toronto, ON Canada; 3 University of Oklahoma, College of Medicine, Oklahoma City, OK USA; 4 The Skin Surgery Center and Department of Plastic and Reconstructive Surgery, Wake Forest Baptist Health, Winston-Salem, NC USA; 5 Dept. of Surgery, Johns Hopkins University, Baltimore, MD USA

**Keywords:** Audit-and-feedback, Process evaluation, Theoretical domains framework, Continuous quality improvement, Mohs micrographic surgery, Dermatology, Knowledge translation

## Abstract

**Background:**

The Improving Wisely intervention is a peer-to-peer audit and feedback intervention to reduce overuse of Mohs Micrographic Surgery (MMS). The objective of this study was to conduct a process evaluation to evaluate Mohs surgeons’ perceptions of the implementation quality and perceived impact of the Improving Wisely intervention.

**Methods:**

Surgeons in the Improving Wisely intervention arm, comprised of members of the American College of Mohs Surgeons (ACMS) who co-led the intervention, were invited to complete surveys and key informant interviews. Participants described perceptions of implementation quality (evaluated via dose, quality of implementation, reach and participant responsiveness), perceived impact of the Improving Wisely intervention (evaluated on a 1–5 Likert and qualitatively), and barriers and facilitators to changing surgeons’ clinical practice patterns to reduce Mohs overuse.

**Results:**

Seven hundred thirty-seven surgeons participated in the survey. 89% were supportive of the intervention. Participants agreed that the intervention would improve patient care and reduce the annual costs of Mohs surgery. Thirty surgeons participated in key informant interviews. 93% were interested in receiving additional data reports in the future. Participants recommended the reports be disseminated annually, that the reports be expanded to include appropriateness data, and that the intervention be extended to non ACMS members. Six themes identifying factors impacting potential MMS overuse were identified.

**Conclusions:**

Participants were strongly supportive of the intervention. We present the template used to design and implement the Improving Wisely intervention and provide suggestions for specialty societies interested in leading similar quality improvement interventions among their members.

**Supplementary Information:**

The online version contains supplementary material available at 10.1186/s12913-020-06017-4.

## Background

Mohs Micrographic Surgery (MMS) is a procedure where dermatologic surgeons aim to obtain a cancer-free margin while preserving healthy tissue by removing a lesion in small slices called stages. This approach is typically used to treat skin cancers with aggressive pathology in areas at high risk for recurrence and/or with functional or cosmetic implications, including tumors on the head and neck, genitals, hands and feet. In the past two decades, the prevalence of MMS use has increased by over 700%, with over 2000 physicians performing Mohs surgery annually in the United States (Chen et al. 2016).

A 2017 study by our research team showed marked variation in the number of stages per case performed for MMS procedures. Approximately 6% of physicians who billed Medicare for MMS between 2012 and 2014 were found to be outliers, defined as surgeons performing a stage per case rate greater than 2 standard deviations from the national mean (Chen et al. 2016; Krishnan et al. 2017).

To address this variation, we developed the Improving Wisely (IW) intervention, a quality improvement initiative that utilizes national data to identify overuse patterns for various medical and surgical procedures. The intervention is composed of personalized, confidential data reports coupled with use of opinion leaders and opportunities for confidential mentoring and education. In a non-randomized controlled trial, 84% of outlier surgeons in the intervention group showed a reduction in mean stages compared to 69% of outliers in the control group.

We posit that the IW intervention can be sustainability implemented to mitigate overuse, underuse or misuse both in Mohs surgery and other areas of medicine. Prior to sustained implementation, we wished to conduct a process evaluation to canvass surgeons’ perceptions on the perceived appropriateness, impact and generalizability of the IW intervention (Moore et al. 2015). Therefore, the purpose of this study was to use mixed methods to evaluate Mohs surgeons’ perceptions regarding the implementation quality and perceived impact of the IW intervention and to identify barriers and facilitators to changing Mohs surgeons’ clinical practice patterns to reduce Mohs overuse.

## Methods

The study design included surveys and key informant interviews to inform a process evaluation for the IW intervention. In Part I, we describe the methods used to design and conduct the survey. In Part II, we use the Consolidated Criteria for Reporting Qualitative Research (COREQ) checklist to report the methods used to design and conduct the key informant interviews (Tong et al. 2007).

### IW Mohs intervention

The Mohs intervention was delivered to 1045 Mohs surgeons who were members of the American College of Mohs Surgery (ACMS), the largest specialty society for MMS. Surgeons received a confidential, personalized report that presented each surgeon’s mean number of stages per case for head and neck lesions, accompanied with the national average of stages per case and a consensus metric definition of acceptable stages per case, defined by an expert panel. Surgeons were categorized as inliers or outliers (i.e., fell outside of the accepted range of variation, defined as > 2.0 standard deviations above the national mean) (Albertini et al. 2019). The confidential reports were endorsed by national opinion leaders at the annual ACMS meeting. The data reports were accompanied with a cover letter which was signed by national opinion leaders. The intervention group were offered opportunities for confidential mentoring and education (Albertini et al. 2019).

In a non-randomized controlled trial, an additional 14% of outlier surgeons in the intervention group decreased their mean stages per case as compared to the control group, which resulted in a statistically significant decrease in mean stages per case across groups. Notified inliers also significantly decreased their stages per case, as compared to non-notified inliers. This study and findings are described in detail elsewhere (Krishnan et al. 2017; Albertini et al. 2019).

### Part I – surveys

#### Setting and participants

The surveys were distributed on May 5, 2018 to ACMS members attending the annual scientific meeting in Chicago. Surveys were distributed during the morning plenary session to facilitate maximum reach of potential participants. All participants who attended the plenary session were invited to complete a survey (regardless of whether or not they had received an IW report). Prior to distribution of the survey, ACMS leaders led a short presentation describing the IW intervention, including a depiction of a sample report. This provided an opportunity for ACMS members who had not received a personalized data report to also provide feedback on their perceptions of the intervention.

#### Outcomes

Outcomes included 1) Perceptions of the IW data; 2) Perceptions of intervention impact; and 3) Perceptions of intervention generalizability and acceptability.

#### Variables

The survey questions were informed by the Theoretical Domains Framework (TDF), which is a framework rooted in psychology theory (Michie and Prestwich 2010; Cane et al. 2012). The TDF includes 14 domains used to elicit perceptions of the factors(barriers and/or facilitators) that can impact physician behavior and can also be used to evaluate a behavioral intervention (Michie and Prestwich 2010; Cane et al. 2012). An initial 35-item questionnaire (2–4 items per TDF domain) was generated. The survey items were iteratively refined and reviewed by knowledge experts (surgeons, ACMS leadership), an implementation scientist and IW team researchers.

The final survey included three categories and 29 questions (Additional File 1). Part 1, Perceptions of the Report Data, included 12 questions. Part 2, Perceptions of Impact, included 9 questions. Part 3, Generalizability and Acceptability, included 8 questions. Data were evaluated using a 5 point, anchored Likert scale (5 = Strongly Agree; 4 = Agree; 3 = Neither Agree nor Disagree; 2 = Disagree; 1 = Strongly Disagree). Negatively and positively worded questions for the same item were included to identify potential response bias (e.g., I *am supportive* vs. *I am not supportive of the IW intervention*). The survey included screening questions to determine the proportion of respondents who were aware of the IW initiative and who received/saw an individualized data report. An open ended question invited participants to highlight other feedback/comments. Demographics collected via the survey included participant name (optional), sex), age range, and years of practice as a Mohs surgeon.

#### Data collection

Surveys were distributed in paper format and were available for online access via RedCap software for a 6 month period (May–October 2018). The survey included a statement indicating that completion of the survey would serve as consent to participation.

#### Data analysis

Descriptive statistics were generated using Excel software. Frequencies and proportions were provided for demographic data. Median, mode and range were reported for Likert data. Missing data were not included in the analysis, total responses per survey question were reported. Positively or negatively framed questions for similar items were examined for response bias (Paulhus 1991). Open-ended data were collated by theme by a single researcher (CF). Frequencies for each theme were generated; individual themes within each comment were counted independently. Themes reported by fewer than < 5 surgeons were excluded, in order to protect confidentiality. Quantitative and qualitative data were triangulated to provide an overall summary of the survey findings (Oleinik 2011).

#### Sample size

The survey was distributed to approximately 900 ACMS Mohs Surgeons attending the annual meeting.

### Part II – key informant interviews

#### Research team and reflexivity

The primary author (CF) conducted the interviews as a postdoctoral fellow on the Improving Wisely research team. The interviewer holds extensive experience conducting key informant interviews. CF did not hold a relationship with the study participants.

#### Study design

##### Theory

The interviews were theoretically rooted in the Theoretical Domains Framework and analyzed using a content analysis approach.

##### Sampling

We aimed to purposively and representatively recruit ACMS members who had received an IW report and were classified as an outlier or an inlier.

##### Recruitment

ACMS Mohs surgeons were emailed a recruitment script inviting them to participate in a 20–30 min telephone interview to describe their overall feedback and perceptions of the IW report and project. Interested participants were asked to respond to the email.

##### Sample Size

We aimed to recruit *n* = 13 outliers and *n* = 13 inliers for a total of *n* = 26 participants. This sample size was informed by the guidance of Francis et al. which suggests using an initial sample size of *n* = 10 and a stopping criteria of *n*= 3 when conducting theoretically-rooted key informant interviews (Francis et al. 2010). We reached out to 267 inliers and 24 outliers, for whom we had an email address. Each surgeon received a personalized email that outlined the study objectives and invited them to participate in a confidential interview. Given the limited sample of outliers, each outlier was sent two recruitment reminders to participate in the key informant interview (Hoddinott and Bass 1986).

##### Data collection

A researcher corresponded with interested participants to schedule the semi-structured interviews. Following verbal informed consent, the interview was recorded and transcribed verbatim.

##### Presence of non-participants

Only the interviewer was present in the room during the interviews.

##### Interview guide

We designed an 11 item semi-structured interview guide (Additional File 2) informed by the TDF and usability questionnaires (Cane et al. 2012; Chisnell and Rubin 2008).

##### Repeat interviews

Key informants participated in a single study interview; transcripts were not returned to the participants.

##### Field notes

The interviewer (CF) took detailed field notes during and following each key informant interview, noting potential themes.

#### Analysis and findings

*Data Analysis:*Summary statistics were generated for demographic variables. Interview recordings were transcribed by an independent agency. Two researchers (CF, WEB) each independently read 3 transcripts to gain familiarity with the interview content. The researchers then developed a codebook based on the TDF (Cane et al. 2012).Additionally, codes were developed to evaluate the implementation quality and ways to improve the IW intervention. Implementation quality, sometimes referred to as a process evaluation, can be used to determine whether the IW intervention was appropriately implemented, or if there were potential barriers that hindered successful implementation (Moore et al. 2015). Process evaluations can explain why an intervention was/was not successful at impacting target outcomes. Implementation quality codes were rooted in the Durlak and Dupre process evaluation framework (Durlak and DuPre 2008) and included*dose* (i.e., how often surgeons want to view the report), *quality of delivery* (i.e., was the report helpful? was it easy to understand/view?), *reach* (i.e., did the right audience receive the report), and *participant responsiveness* (i.e., were surgeons engaged in/approving of the IW intervention?).

Definitions and coding rules were developed for each TDF domain and process measure. The researchers double coded 35% of the interviews using NVivo software (Nvivo 1999). Any discrepancies were resolved viaconsensus discussion. The remainder of the interviews were divided and each half was single coded by one researcher. The codes were reviewed by the researchers to develop a list of overarching themes.

## Results

### Survey results

#### Demographics

A total of 737 ACMS Mohs surgeons participated in the survey (response rate = 82%). Participants were mostly male (62%) and between the ages of 30–49 (68%). Ninety-six percent of those surveyed were aware of the IW intervention and 76% had received an individualized feedback report. Among those who received a report, 88% reported their mean number of stages were ‘as they had expected’; 7 and 6% reported their mean number of stages were lower and greater than expected, respectively (Table 1). 89% were supportive of the intervention.

**Table 1:** Participant demographics and Summary Responses

SURVEY PARTICIPANTS			INTERVIEW PARTICIPANTS		
Item	n	%	Item	n	%
**Sex**	Male (453) Female (256) Prefer not to answer (28)	61.47 34.74 3.80	**Sex**	Male (19) Female (11)	63% 37%
**Age**	< 30 (1) 30–39 (237) 40–49 (262) 50–59 (138) 60–69 (65) 70+ (13) Prefer not to answer (21)	0.14 32.16 35.55 18.72 8.82 1.76 2.85	**Years of Practice as Mohs surgeon**	0–5 (2) 6–10 (13) 11–15 (4) 16–20 (3) 21–30 (5) 31–40 (1) 41+ (0) Prefer not to answer (2)	6.66 43.33 13.33 10.00 16.66 3.33 0.00 6.67
**Years of Practice as Mohs surgeon**	0–5 (205) 6–10 (154) 11–15 (121) 16–20 (85) 21–30 (109) 31–40 (44) 41+ (4) Prefer not to answer (15)	27.82 20.90 16.42 11.53 14.79 6.00 0.54 2.03	**Outlier/Inlier Status**	Notified Outlier (4) Notified Inlier (26)	13.33 86.66
**Aware of the Improving Wisely intervention**	Yes (700) No (15) Unsure (13) No response (9)	96.15 2.06 1.79	**Practice Type**	Academic/Public (8) Private (22)	26.66 73.33
**Have received an Improving Wisely data report**	Yes (551) No (153) Unsure (21) No response (9)	75.69 21.02 2.88			
**Expectations of mean number of stages as described in data report**	As I had expected (476) Greater than I expected (30) Lower than I expected (38) No response/ NA (193)	87.50 5.51 6.99			
**Preferred frequency of Improving Wisely intervention**	Annually (483) Biannually (162) Quarterly (35) Never/ Not Needed (8) No response (49)	70.20 23.55 5.09 1.16			

%calculation- missing data excluded from denominator

#### Part 1: perceptions of intervention 

The majority of participants found the IW intervention simple to interpret and expressed strong interest in receiving another utilization report in the future (median, mode = 5). Participants strongly agreed that it was valuable to see their data compared to national benchmarks and that it was personally important to know that their stages fell within the ACMS-established boundaries of variation. Despite support for the intervention, seeing the data reports did not change surgeon’s perceptions of their practice patterns (Table 2).

**Table 2:** Survey Results

Item	TDF Domain	Median, mode (range 1–5)		N (total responses per question)
		**PART 1: Perceptions of intervention**		
		1 – Strongly Disagree; 2 – Disagree; 3 – Neither Agree nor Disagree; 4 – Agree; 5 – Strongly Agree		
1	Knowledge	Seeing my report has changed my perception of my practice patterns	3, 3	613
2	Knowledge	I would be interested in seeing my utilization report again in the future	5, 5	659
3	Skills	It is simple to interpret the results of the Improving Wisely report	5, 5	631
4	Social/Professional Role/Identity	It is important for me that my performance falls within the boundaries of variation established by ACMS (1.1–2.2 stages per case)	5, 5	667
5	Social/Professional Role/Identity	I find value in seeing my procedure data compared to national benchmarks	5, 5	668
6	Beliefs about Consequences	I am concerned this report could negatively impact my reputation, even though it is confidential	2, 2	661
7	Believes about Capabilities	I am confident that I can achieve (outlier) / can continue to achieve (non-outlier) a practice pattern within the national average within the next 6 months	5, 5	656
		**PART 2: Perceptions of Impact**		
8	Optimism	I believe the Improving Wisely project will improve patient care	4, 5	690
9	Optimism	I believe the Improving Wisely project will reduce annual costs of Mohs surgery to the system	4, 5	689
10	Optimism	I believe that the Improving Wisely project will change Mohs surgeons’ behaviour	4, 4	688
11	Optimism	I believe that improvements in surgeon practice due to Improving Wisely will be sustained long term **with** repeat reports	4, 4	688
12	Optimism	I believe that improvements in surgeon practice due to Improving Wisely will be sustained long term **without** repeat reports	2, 2	686
13	Beliefs about Consequences	The Improving Wisely report made me more aware of unnecessary medical care	3, 4	666
14	Beliefs about Consequences	The Improving Wisely project has improved the quality of my surgical practice	3, 3	647
15	Intentions	After seeing my individual data, I intend to be more mindful of my stages per case rate	3, 3	638
16	Memory, Attention and Decision Processes	Knowledge of my procedure data influence the way I perform Mohs surgery	3, 3	649
		**PART 3: Overall Impressions**		
17	Environmental context and resources	The principles of Improving Wisely make sense in the context of Mohs surgery	5, 5	683
18	Environmental context and resources	I believe the Improving Wisely data sharing approach can be valuable for other areas of medicine	5, 5	682
19	Social Influences	Most people whose opinions I value would support the Improving Wisely project	5, 5	676
20	Social/Professional Role/Identity	The goals of Improving Wisely project should be communicated to trainees	5, 5	682
21	Emotion	I felt undue pressure from seeing my data report	2, 2	646
22	Emotion	I feel threatened by this project	2, 1	667
23	Optimism	I am supportive of the Improving Wisely initiative	5, 5	676
24	Optimism	I do **NOT** support the Improving Wisely project	1, 1	667

#### Part 2: perceptions of impact

Participants agreed or strongly agreed that the IW intervention would improve patient care and reduce annual costs of Mohs surgery (Table 2). Participants agreed that improvements in surgeon practice would be sustained with repeated, annual reports. Participants’ gave neutral responses regarding beliefs about personal consequences, intentions, and decision making processes (e.g., *the report has improved the quality of my surgical practice; I intend to be more mindful of my stages per case rate*).

#### Part 3: overall impressions and generalizability of the intervention

The majority of participants believed that the principles of the IW intervention would also be valuable for other areas of medicine (median, mode = 5). Participants agreed that their peers would support the IW project and suggested that the intervention goals be communicated to Mohs trainees. Participants did not feel threatened by the project (median, mode = 2,1) or experience undue pressure from seeing their report (median, mode = 2).

#### Open ended feedback data

Analysis of the open-ended survey data revealed three overarching categories and 7 themes, outlined alongside participant quotes in Additional File 3. The first category (3 themes) presented feedback on the data used to create the report. *N* = 23 participants (3%) expressed concern that complex cases may be driving up their average. Commonly, case complexity was attributed to working in a tertiary referral center or in a rural area. Less than 1% of participants (*n* = 6) suggested that specific appropriateness criteria, rather than statistical variation, should be used to determine outlier status in order to ensure a sustainable quality improvement intervention. *N* = 35 participants (5%) suggested additional metrics for IW reports; common suggestions included flaps versus grafts and next day closures.

The second category (3 themes) of feedback was categorized as general support for the IW intervention. Five participants (< 1%) reported that the data report made them more mindful of stages per case, the cost of health care, and highlighted that they share this metric with their patients. Seven participants (1%) suggested the reach of the intervention should be extended to include every provider that bills Mohs codes (i.e., do not limit to ACMS members). Finally, 11% of participants provided positive feedback on the intervention and praise for the ACMS for leading this intervention.

The third category (1 theme) highlighted concerns about the potential unintended consequences of the IW intervention. *N* = 29 (4%) were concerned that these data would no longer be confidential (i.e., might get ‘leaked’ to policymakers, funders or patients). Some participants expressed concerns that surgeons might change their practice to cater to the report rather than patient care (e.g., take unnecessarily large margins to reduce their mean score). Others suggested that outliers, once aware that this metric was being tracked, would continue to ‘game’ the system in other ways.

#### Triangulation

The responses did not indicate evidence of a response bias. Polarized scores were demonstrated for negatively and positively worded questions for the same item (see Table 2, items 11 and 12, 23 and 24). The quantitative and open-ended survey data were convergent.

### Part 2 – key informant interviews

Thirty Mohs surgeons participated in the key informant interviews. The average length of the interviews was 21 min (range: 10–50 min.). Interviews were conducted between September 12, 2018-January 24, 2019. Sixty-three percent of interview participants were male and 43% had 6–10 years of practice as a Mohs surgeon. A total of 26 notified inliers (87%) and 4 notified outliers (13%) participated in the interviews. Saturation was reached for the inlier, but not the outlier group.

#### Process evaluation of IW intervention

##### Dose

93% of those interviewed were interested in seeing their data report in the future. Consistent with the surveys, the majority agreed that the report should be disseminated annually, suggesting that this frequency would be sufficient to nudge outliers without creating too much burden (See Table 3 for sample quotes).

**Table 3:** Implementation Quality – Key Informant Data, Sample Quotes

Domain/Category	Theme	Quote	Participant ID, status
Dose	Deliver Reports Annually	*I think just continuing the program, so you don’t get it [the report] once every decade or once every 5 years, it’ll maybe remind the outliers ‘Oh, they’re still out there'*	P5, inlier
Quality of Delivery	Report was easy to read, understand	*I thought it was very intuitive, it’s done incredibly well and I think the data is so critical*	P4, inlier
	Expand report to include non- CMS data	*I have Medicare patients, I operate in a Veterans hospital which is not included. And my privately insured patients were not included.*	P9, inlier
	Include appropriateness use metrics to strengthen data credibility	*I’m afraid [without appropriateness use criteria] we end up trying to create a top-hat type curve. Then we end up with a very, very narrow window of what is ‘acceptable’ for number of players*	P11, inlier
Participant Responsiveness	Reassurance of being within the mean	*I really enjoyed looking at the data and seeing how I compared to other surgeons.* *You leave your fellowship and nobody is there anymore watching what you do…and so every now and then it’s so nice to just have like some sort of data saying, ‘hey, here’s what you’re doing compared to everyone else, and it seems to be like you’re A-ok, you know?’*	P22, inlier P2, inlier
	Support for ACMS as quality improvement leaders	*Things like this are only going to benefit the community of Mohs surgeons..the Mohs College is at the leading edge of doing this. I think it shows that it’s what’s going to differentiate us from people who have not done an accredited Mohs surgery fellowship*	P14, inlier
	Belief that it’s better to ‘police your own’	*We can make changes in our own practices before a regulated body, the government for example, comes and says you know ‘you’re making too much money…we’re going to cut the reimbursement; if we can make changes from within, then we [can] protect ourselves from outside scrutiny*	P6, inlier
Reach	Perception that outlier problem lies with non-college members	*In my opinion, I suspect that a lot of the overuse of Mohs surgery codes may be practiced by folks who are not members of the Mohs college…maybe we can be looking at it across a specialty – dermatology- and not just Mohs College members.*	P7, inlier
Perceptions of Impact	Improved Awareness of Overuse	*I think that it’s natural to become more mindful of something if you know that data is being recorded on it.*	P5, inlier
	Potential Unintended Consequences - Gaming - Undue pressure - Compromised patient quality	*We have purposefully taken measures to try and minimize our number of levels taken or our stages to the detriment of our patients on many occasions* *[Improving Wisely] is a great awareness tool that should [help] people change their practice for the better, or it will make them hide the bad things they’re doing, hide them deeper* *Just the other day I did six stages on a patient and I was like ‘you’re killing me’. Like I’m so aware of this, it kills me now. When that happens, it’s like oh my God, this tumor is gonna mess me up*	P28, outlier P1, outlier

##### Quality of Delivery

All interviewed participants found the report straight forward, easy to interpret and clear (Table 3). Suggestions on how to improve the report included expanding the data report to include non-CMS databases (e.g., private insurance) and incorporating appropriate use criteria to establish recommended targets. Participants believed that the incorporation of appropriateness would detect differences in geographic location or case complexity.

##### Participant responsiveness

Participants, particularly notified inliers, were generally supportive of the IW intervention. The most common identified theme was the sense of reassurance generated by the report. Participants were supportive of ACMS leading the IW intervention, highlighting the importance of ‘policing your own’ before another regulatory body did so (Table 3).

##### Reach

Many participants suggested that the intervention should be extended to non-ACMS members.

##### Perceptions of Impact

The most common theme regarding perceived impact of the intervention was that it improved surgeon awareness of Mohs overuse (Table 3). Some participants, particularly those classified as outliers, were concerned that the intervention might have unintended consequences, such as gaming the system, adding undue pressure, or compromising patient quality.

#### Factors impacting Mohs overuse

Six main themes, representing six TDF domains that impact Mohs overuse were identified. The most common theme was the perception that financial incentives drive Mohs overuse (Table 4). Tied closely to the facilitator of financial incentives was the perception that there was a lack of regulation. Both inlier and outlier participants highlighted that lack of monitoring led to either the conscious or unconscious justification of taking more stages per case. Another common theme, particularly highlighted by participants classified as outliers, is that geographic variation or clinic type impacts the types of cases presenting for Mohs surgery. Because these cases tend to be more complex, or because there is less access to Mohs care in rural areas, more stages per case are required in order to provide an adequate treatment. Participants who highlighted this theme were supportive of incorporating appropriate use criteria into the IW reports. Finally, the last two themes pertained to surgeon experience, beliefs about capabilities, and surgical skill. Both inlier and outlier surgeons reported that skills ‘slip’ once surgeons leave fellowship training. Additionally, participants hypothesized that inexperienced or less confident surgeons may err on the side of caution, opting to take additional stages to ensure patient safety.

**Table 4:** Factors impacting potential Mohs overuse

TDF Domain	Theme	Quote	Participant ID, status
Behavioral Regulation	Overuse driven by lack of regulation/monitoring	*If you’re not being audited or you’re not being monitored…then they [surgeons] could justify doing these things to increase their outcome or offset expenses and they may do it again, consciously or subconsciously. I don’t think its an overnight thing* *He [colleague] would automatically take two layers with every single patient, whether it had a positive margin or not…we reported him to [regulatory body] many times…the person next door is doing it. – P4, inlier*	P1, outlier P4, inlier
Reinforcement	Financial incentives driving overuse	*The easiest way to cheat is to just take an extra layer. You can easily justify it to Medicare or the third party insurance. And extra layers are reimbursed you know at 100%, so totally inappropriate use of Mohs goes on all the time* *When you’re in fellowship, you’re not necessarily aware of reimbursement issues and so when you’re in more private practice, you’re more keenly aware of reimbursement and numbers so again, consciously or subconsciously, you make decisions that make you more money*	P8, inlier P2, inlier
Beliefs about Capabilities	Required improvements in education, training and confidence Inexperience	*If I remember back to when I was just out of my training, I may have been overly cautious…and ended up with extra stages.* *There’s been a proliferation of very young Mohs surgeons as there become more training programs. Maybe they’re a little bit less comfortable with their reconstructive skills and afraid to make a too large hole*	P18, inlier P4, inlier
Skills	Surgeons stray from original training/ skills need to be re-developed	*I feel that I slipped in the way that I do surgery…I felt like okay, what am I doing, like am I doing flaps all the time, am I doing linear complex repairs? You know, a fish doesn’t know its swimming in muddy water* *I think part is quality and training. I think a lot of people see a little bit of inflammation and they’re like, ‘oh there’s a tumor here, let me take it out just in case’ and they get another stage out of it*	P1, inlier P3, outlier
Environmental context and resources	Geographic Variation, Clinic Type might impact typical case complexity/ number of stages	*I have a low number of stages because I live in Florida. Even though there’s a ton of skin cancer, people have a high utilization of dermatology. They’re plugged in.* *We’re the only major Mohs [clinic] in [location retracted to protect clinic anonymity]. So we have patients that come from [many other regions] because we’re experienced*	P3, inlier P8, inlier
Social, Professional Role/Identity	Practicing defensively for patient safety/cosmetic result may lead to increased number of stages	*If you more heavily weigh the cosmetic aspect, you’re probably going to be taking more layers per case*	P2, inlier

Most interview participants, including outliers interviewed, were supportive of the IW intervention. Nearly all participants highlighted that the data report made them more aware of their stages per case. Of the four outliers interviewed, three attributed their outlier status to having more complex cases or being in an underserviced geographic location. One believed that financial gain was driving up overuse of Mohs procedures.

The most common suggestion to reduce Mohs overuse was the use of ‘champions’. The suggested role of these champions is to support decision making for complex cases (e.g., peer review) and to reach out to ‘coach’ identified outliers in a supportive manner.

## Discussion

We report a process evaluation for the IW intervention, aimed at reducing potential overuse of Mohs surgery. Surveys with Mohs surgeons revealed strong majority support for the intervention, and provided useful suggestions on additional metrics. Interviews with Mohs surgeons provided feedback on the preferred delivery of the data reports and confirmed the acceptability and utility of the IW intervention. Participants most commonly believed that Mohs overuse was driven by financial incentives and surgeon inexperience. Interview participants also stressed the importance of incorporating Mohs appropriate use criteria to generate future data reports and to account for potential geographic variation or case complexity.

This study demonstrates the value of conducting process evaluations for implementation interventions. Increasingly, implementation researchers have called for improved standards for tracking and reporting process evaluations to support widespread implementation and sustainability (Lewis et al. 2018). Process evaluations provide researchers and implementers with insight on why an intervention may have worked, or not; illuminates potentially critical barriers and facilitators that can aid in further implementation; and supports an integrated approach of involving the user in the evaluation of the implementation, which may lead to improved uptake and sustainability (Limbani et al. 2019).

The IW intervention is a relatively low cost intervention that has resulted in significant clinical and positive process outcomes (Albertini et al. 2019). We posit the success of the IW intervention can be attributed to three main factors. First, the data reports are rooted in best practices for audit and feedback, including recommending specific actions, providing individualized data, using comparators to reinforce suggestions, presenting data both visually and in text while reducing cognitive overload, and by preventing negative reactions to the feedback by emphasizing the non-punitive, collaborative approach of the intervention (Brehaut et al. 2016). Second, we use an integrated knowledge translation approach to developing the datareports, meaning that content experts in the field lead, create and endorse the metrics used to generate the data reports (Gagliardi et al. 2015; Graham et al. 2018). This approach is believed to improve both the buy-in for and impact of implementation strategies (Gagliardi et al. 2015; Graham et al. 2018). Finally, the intervention is endorsed by respected leadership and opinion leaders (Flodgren et al. 2019). We hypothesize that the feasibility and the impact of the IW intervention would have been compromised without the support of ACMS leadership.

**Fig. 1:**
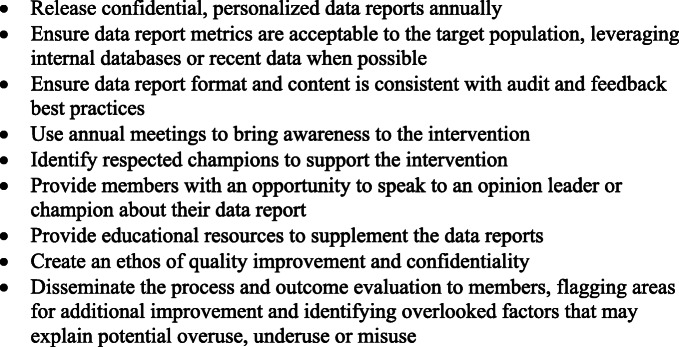
Summary of recommendations

The IW intervention was first piloted among Mohs surgeons. Recently, our team has expanded the intervention to address other areas of overuse, underuse and misuse in medicine and surgery (ClinicalTrials.gov. 2019). Interestingly, one of our most significant barriers to spread of the IW intervention is our ability to engage specialty societies to endorse, design and co-deliver the intervention. We are optimistic that the positive feedback presented in this study may promote society engagement and provide an example of how specialty societies can take a leadership role in leading quality improvement initiatives among their members. We provide in Fig.1 a summary of key recommendations based on our experience that may be generalizable to other specialty societies interested in implementing similar interventions.

Finally, this study is not without limitations. First, we were unable to successfully recruit our target sample size of outlier surgeons, despite significant recruitment efforts. Given that outliers are the primary target of the IW intervention, not having their voice fully represented presents a significant gap in knowledge, and engaging this population presents an area for additional research. We posit the low recruitment rate may be attributed to a limited population sample, which may not have been sufficient to recruit our target of *n* = 13. Our team contacted *n* = 24 outliers who received an IW report, meaning we required a > 50% recruitment rate to reach the *n* = 13 target. Additionally, it is possible that outliers did not agree with the data report findings. 75% of the outliers we interviewed suggested that geographic variation or case complexity were the factors influencing their increased Mohs utilization. Further integration of Mohs appropriate use criteria into the data metrics may result in increased buy-in, particularly among outlier surgeons. Second, it is likely that our sample population, namely Mohs surgeons who are members of a leading specialty society and who have attended the ACMS annual meeting, represent a sample of physicians who are potentially more supportive of society-led quality improvement interventions. We are currently in the process of conducting another IW trial that will deliver feedback interventions to both specialty society members and non-members. Collation of participant feedback to this iteration will provide an interesting comparison to this study.

## Conclusions

The majority of study participants supported the Improving Wisely peer-to-peer, audit and feedback intervention; participants were supportive of receiving additional data reports in future. Participants provided informative feedback on the content and structure of data reports that should be incorporated in future iterations of the intervention. Six themes identifying factors impacting potential MMS overuse were identified; additional interventions can be used to address these factors. Specialty societies can play an important role to ensure appropriate use of surgical, clinical and diagnostic procedures. We present the template used to design and implement the Improving Wisely intervention as an example for societies who are interested in leading initiatives to improve appropriate use of medications, procedures or diagnostic testing among their members. Finally, this study demonstrates the value of conducting process evaluations for implementation research and supports the use of an integrated approach to involving the target user in implementation evaluation.

## Supplementary Information

**Additional file 1.** Survey

**Additional file 2.** Interview Guide

**Additional file 3.** Open-ended Survey Data

## Data Availability

The datasets used and/or analysed during the current study are available from the corresponding author on reasonable request.

## Abbreviations

ACMS: American College of Mohs Surgery
IW: Improving Wisely Intervention
MMS: Mohs Micrographic Surgery
TDF: Theoretical Domains Framework
