# Supplementary material for: A process evaluation of the improving wisely intervention: a peer-to-peer data intervention to reduce overuse in surgery
Source: BMC Health Serv Res. 2021 Jan 29;21:100. doi: 10.1186/s12913-020-06017-4 (PMC7845024; doi:10.1186/s12913-020-06017-4)
Supplement: Supplementary file 1 — Additional file 1. Survey [file 12913_2020_6017_MOESM1_ESM.docx]

Additional File 1: Survey

*Dear Participant,*

*You are invited to participate in this research study with Johns Hopkins because you are a Mohs surgeon who has received an Improving Wisely report. The purpose of this survey is to seek information about your overall thoughts and perceptions of the Improving Wisely project. Your participation in this survey is voluntary. You may choose not to participate. If you decide to participate in this survey, you may withdraw at any time. Your answers will be kept confidential. Responses will be reported in aggregate format. We really appreciate your willingness to participate and value your feedback. For more information on this study please contact Dr. Christine Fahim at* [*cfahim1@jhu.edu*](mailto:cfahim1@jhu.edu)*. Dr. Martin Makary (*[*mmakary1@jhmi.edu*](mailto:mmakary1@jhmi.edu)*) is the study PI.*

***Your completion of this survey will serve as your consent to be in this research study.***

*Thank you,
Martin Makary, Christine Fahim, and John Albertini*

**Name _____________________________________________**Names used only to keep track of responses and will remain confidential. Data will be anonymous. Write ‘anonymous’ if you do not wish to give your name.

**Sex** ☐ Male ☐ Female **Age** ☐<30 ☐30-39 ☐40-49 ☐50-59 ☐60-69 ☐70+ yrs

**Yrs. of Practice** ☐0-5 ☐6-10 ☐11-15 ☐16-20 ☐21-30 ☐31-40 ☐41+

I am aware of the quality improvement initiative “Improving Wisely” to encourage best practices around **☐Yes ☐No ☐Unsure**

utilization (e.g., mean stages per Mohs case)

I have seen my Improving Wisely data report showing my mean stages per case in comparison to my **☐Yes ☐No ☐Unsure**

Colleagues

My mean number of stages per case were **☐As I had expected ☐Lower than expected ☐Greater than expected**

How often do you think the Improving Wisely data reports should be disseminated? **☐Never ☐Quarterly ☐Biannually ☐Annually**

| **PART 1: Perceptions of Data** | | | | | |
| --- | --- | --- | --- | --- | --- |
| **1 –** Strongly Agree**; 2 –** Agree**; 3 –** Neither Agree nor Disagree; **4 –** Disagree**; 5 –** Strongly Disagree | | | | | |
| Seeing my report has changed my perception of my practice patterns | 1 | 2 | 3 | 4 | 5 |
| I would be interested in seeing my utilization report again in the future | 1 | 2 | 3 | 4 | 5 |
| It is simple to interpret the results of the Improving Wisely report | 1 | 2 | 3 | 4 | 5 |
| It is important for me that my performance falls within the boundaries of variation established by ACMS (1.1-2.2 stages per case) | 1 | 2 | 3 | 4 | 5 |
| I find value in seeing my procedure data compared to national benchmarks *Survey continues on back of page* | 1 | 2 | 3 | 4 | 5 |
| I am concerned this report could negatively impact my reputation, even though it is confidential | 1 | 2 | 3 | 4 | 5 |
| I am confident that I can achieve (outlier) / can continue to achieve (non-outlier) a practice pattern within the national average within the next 6 months | 1 | 2 | 3 | 4 | 5 |
| **PART 2: Perceptions of Impact** | | | | | |
| **1 –** Strongly Agree**; 2 –** Agree**; 3 –** Neither Agree nor Disagree; **4 –** Disagree**; 5 –** Strongly Disagree | | | | | |
| I believe the Improving Wisely project will improve patient care | 1 | 2 | 3 | 4 | 5 |
| I believe the Improving Wisely project will reduce annual costs of Mohs surgery to the system | 1 | 2 | 3 | 4 | 5 |
| I believe that the Improving Wisely project will change Mohs surgeons’ behaviour | 1 | 2 | 3 | 4 | 5 |
| I believe that improvements in surgeon practice due to Improving Wisely will be sustained long term **with** repeat reports | 1 | 2 | 3 | 4 | 5 |
| I believe that improvements in surgeon practice due to Improving Wisely will be sustained long term **without** repeat reports | 1 | 2 | 3 | 4 | 5 |
| The Improving Wisely report made me more aware of unnecessary medical care | 1 | 2 | 3 | 4 | 5 |
| The Improving Wisely project has improved the quality of my surgical practice | 1 | 2 | 3 | 4 | 5 |
| After seeing my individual data, I intend to be more mindful of my stages per case rate | 1 | 2 | 3 | 4 | 5 |
| Knowledge of my procedure data influence the way I perform Mohs surgery | 1 | 2 | 3 | 4 | 5 |
| **PART 3: Overall Impressions** | | | | | |
| **1 –** Strongly Agree**; 2 –** Agree**; 3 –** Neither Agree nor Disagree; **4 –** Disagree**; 5 –** Strongly Disagree | | | | | |
| The principles of Improving Wisely make sense in the context of Mohs surgery | 1 | 2 | 3 | 4 | 5 |
| I believe the Improving Wisely data sharing approach can be valuable for other areas of medicine | 1 | 2 | 3 | 4 | 5 |
| Most people whose opinions I value would support the Improving Wisely project | 1 | 2 | 3 | 4 | 5 |
| The goals of Improving Wisely project should be communicated to trainees | 1 | 2 | 3 | 4 | 5 |
| I felt undue pressure from seeing my data report | 1 | 2 | 3 | 4 | 5 |
| I feel threatened by this project | 1 | 2 | 3 | 4 | 5 |
| I am supportive of the Improving Wisely initiative | 1 | 2 | 3 | 4 | 5 |
| I do **NOT** support the Improving Wisely project | 1 | 2 | 3 | 4 | 5 |
