# Supplementary material for: A process evaluation of the improving wisely intervention: a peer-to-peer data intervention to reduce overuse in surgery
Source: BMC Health Serv Res. 2021 Jan 29;21:100. doi: 10.1186/s12913-020-06017-4 (PMC7845024; doi:10.1186/s12913-020-06017-4)
Supplement: Supplementary file 2 — Additional file 2. Interview Guide [file 12913_2020_6017_MOESM2_ESM.docx]

Additional File 2: Interview Guide

1. The first question is regarding our quality metric. Do you believe that some Mohs surgeons are using too many stages per case for head and neck lesions?
   1. If yes, why do you think this is happening? Why is there overuse in this area?
2. Could you tell me what you know about the Improving Wisely project?
3. Have you seen your Improving Wisely data report, showing your mean stages per case?
   1. Did you find the report easy to interpret?
   2. Were your mean number of stages the number you had expected (greater, lower, as expected)
   3. Has the report changed your practice patterns? How?
      1. What triggered you to change your practice patterns? (seeing the report, patient care, colleague opinions of the report)
   4. Would you be interested in seeing your utilization report again in the future?
   5. Were you aware there were other interventions (education, retraining) being offered by the ACMS? Did you seek out any of these interventions?
   6. Any interventions not offered but should have been?
4. Do you value seeing your procedure data compared to national benchmarks?
   1. Has knowledge of your procedure data influenced the way you perform Mohs surgery? If yes, how?
   2. OUTLIERS: Do you believe that your practice patterns will fall within the national average range in the next report? Why or why not?
   3. Did you experience any undue pressure from seeing your report?
5. Do you think the Improving Wisely approach (setting boundaries of expected variation to identify outliers) applies to Mohs surgery? Why or why not?
   1. Are you supportive of this approach? Why or why not?
6. In your opinion, does the Improving Wisely approach encourage surgeons to be more mindful of their stages per case?
   1. Does the report make you more aware of unnecessary medical care?
7. In your opinion, does the Improving Wisely approach improve patient care? Quality of surgical practice?
   1. Do you think any positive changes will be sustainable? If yes, are annual audit reports required to sustain these effects?
8. Do you believe the Improving Wisely approach can reduce health care costs?
   1. If yes, how much would you estimate would be saved by using Mohs more sparingly?
9. Overall, are you supportive of the Improving Wisely approach? Why or why not?
   1. How can we optimize the approach?
   2. Are additional interventions (beyond data reports) required?
      1. If yes, what type of interventions do you think would be most effective?
10. Do you believe that your colleagues support the Improving Wisely project? Why or why not?
