# Supplementary material for: A process evaluation of the improving wisely intervention: a peer-to-peer data intervention to reduce overuse in surgery
Source: BMC Health Serv Res. 2021 Jan 29;21:100. doi: 10.1186/s12913-020-06017-4 (PMC7845024; doi:10.1186/s12913-020-06017-4)
Supplement: Supplementary file 3 — Additional file 3. Open-ended Survey Data [file 12913_2020_6017_MOESM3_ESM.docx]

Additional File 3: Open-ended Survey Data

| **Category** | **Theme** | **Frequency n** | **Sample quotes** |
| --- | --- | --- | --- |
| Feedback regarding data used to create report | Concern that complex cases may be driving up stages per case | 23 | *…we tend to get more difficult tumors due to our reputation which might also factor in – P20*  *I am taking care of complex cases in a more rural area…I think the goals of the project are good and important BUT worry I will be penalized when compared to lower-risk Mohs surgeons – P78*  *I do believe that being in a tertiary referral center places providers at a disadvantage - P418* |
|  | Concerns about future direction of data reports/ metric calculation | 6 | *I’m not sure that being a non-outlier means people are providing better care – P69*  *Statistically by this method, there will always be outliers – P488* |
|  | Suggestions for additional metrics | 35 | *Somebody should take a good look at Mohs surgeons performing closures, especially next day closures –P728*  *Correlate number of stages with number of flaps and number of secondary cases – P32*  *Look at utilization, overutilization of flap closures – P40* |
| Support for the Improving Wisely Intervention | Positive Feedback/ Reinforcement | 82 | *This is an absolutely necessary project. Thank you for this work. – P31*  *We need to decrease unnecessary Mohs layers. Data will improve our specialty. – P80*  *…I do appreciate hearing/knowing the ranges of my colleagues and national averages to have data to compare to – P251* |
|  | Perceptions of Impact | 5 | *After seeing my individual data, I intend to be more mindful of my stages per case rate…[this shouldn’t] be driver of fitting within the average when it comes to treating an individual patient but should be at least part of your mindset – P65*  *Appreciate this work! Thank you!! Already mindful of decreasing cost of health care, so glad to see we are educating our Mohs college. This is the right way to go! – P73*  *I love sharing this metric with patients! –P276* |
|  | Extend reach of intervention | 7 | *Need to send these data reports to every provider that bills Mohs codes (not just to ACMS members) – P85*  *Non college members should be notified in a similar way – P199*  *It would be interesting to compare ACMS versus non-ACMS members – P327* |
| Concerns about the Improving Wisely Intervention | Concerns about unintended consequences (e.g., financial/privilege implications, surgeons gaming to be an ‘inlier’) | 29 | *I am concerned that these reports could intimidate some members to take fewer stages or overly large stages which may actually be a disservice to patients – P288*  *This will be used to hopefully only identify outliers but data might get leaked [and] be used against us – P376*  *I feel that the data is useful but I fear that people will take large margins just to improve their scores – P543* |
